# Association of Infectious Mononucleosis in Childhood and Adolescence With Risk for a Subsequent Multiple Sclerosis Diagnosis Among Siblings

**DOI:** 10.1001/jamanetworkopen.2021.24932

**Published:** 2021-10-11

**Authors:** Yin Xu, Ayako Hiyoshi, Kelsi A. Smith, Fredrik Piehl, Tomas Olsson, Katja Fall, Scott Montgomery

**Affiliations:** 1 Clinical Epidemiology and Biostatistics, School of Medical Sciences, Örebro University, Örebro, Sweden; 2 Department of Public Health Sciences, Stockholm University, Stockholm, Sweden; 3 Department of Epidemiology and Public Health, University College London, London, United Kingdom; 4 Clinical Epidemiology Division, Karolinska Institute, Stockholm, Sweden; 5 Department of Clinical Neuroscience, Karolinska Institute, Stockholm, Sweden; 6 Integrative Epidemiology, Institute of Environmental Medicine, Karolinska Institute, Stockholm, Sweden

## Abstract

**Importance:**

Epstein-Barr virus and its acute manifestation, infectious mononucleosis (IM), are associated with an increased risk of multiple sclerosis (MS). Whether this association is confounded by susceptibility to infection is still debated.

**Objective:**

To assess whether hospital-diagnosed IM during childhood, adolescence, or young adulthood is associated with subsequent MS diagnosis independent of shared familial factors.

**Design, Setting, and Participants:**

This population–based cohort study used the Swedish Total Population Register to identify individuals born in Sweden from January 1, 1958, to December 31, 1994. Participants aged 20 years were followed up from January 1, 1978, to December 31, 2018, with a median follow-up of 15.38 (IQR, 8.68-23.55; range, 0.01-40.96) years. Data were analyzed from October 2020 to July 2021.

**Exposure:**

Hospital-diagnosed IM before 25 years of age.

**Main Outcomes and Measures:**

Diagnoses of MS from 20 years of age were identified. Risk of an MS diagnosis associated with IM in childhood (birth to 10 years of age), adolescence (11-19 years of age), and early adulthood (20-24 years of age [time-dependent variable]) were estimated using conventional and stratified (to address familial environmental or genetic confounding) Cox proportional hazards regression.

**Results:**

Of the 2 492 980 individuals (1 312 119 men [52.63%] and 1 180 861 women [47.37%]) included, 5867 (0.24%) had an MS diagnosis from 20 years of age (median age, 31.50 [IQR, 26.78-37.54] years). Infectious mononucleosis in childhood (hazard ratio [HR], 1.98; 95% CI, 1.21-3.23) and adolescence (HR, 3.00; 95% CI, 2.48-3.63) was associated with an increased risk of an MS diagnosis that remained significant after controlling for shared familial factors in stratified Cox proportional hazards regression (HRs, 2.87 [95% CI, 1.44-5.74] and 3.19 [95% CI, 2.29-4.46], respectively). Infectious mononucleosis in early adulthood was also associated with risk of a subsequent MS diagnosis (HR, 1.89; 95% CI, 1.18-3.05), but this risk was attenuated and was not significant after controlling for shared familial factors (HR, 1.51; 95% CI, 0.82-2.76).

**Conclusions and Relevance:**

These findings suggest that IM in childhood and particularly adolescence is a risk factor associated with a diagnosis of MS, independent of shared familial factors.

## Key Points

**Question:**

Is diagnosis of infectious mononucleosis (IM) in childhood or adolescence associated with subsequent development of multiple sclerosis (MS)?

**Findings:**

In this population–based cohort study of 2 492 980 individuals in Sweden, IM in childhood and adolescence was associated with an increased risk of a subsequent MS diagnosis that remained significant after controlling for measured and unmeasured shared familial factors.

**Meaning:**

These findings suggest that IM in childhood and particularly adolescence is a risk factor for a subsequent MS diagnosis, independent of shared familial factors, making it less likely that greater susceptibility to infection is the explanation.

## Teaser

This cohort study assesses the association of hospital-diagnosed infectious mononucleosis in childhood and adolescence with risk of a multiple sclerosis diagnosis in young adults using national population registers in Sweden.

## Introduction

Infectious mononucleosis (IM) is a clinical manifestation of viral infection, predominantly caused by Epstein-Barr virus (EBV). Both IM and high levels of anti-EBV nuclear antigen 1 (EBNA-1) antibodies are associated with an increased risk for multiple sclerosis (MS).^1,2^

However, the nature of the association between IM and MS is still debated.^3^ This association might be explained, at least in part, by common genetic factors relevant to the acute manifestation of IM, subsequent antibody levels, and MS risk. Genome-wide association studies have found that loci in the human leukocyte antigen (HLA) region, such as *HLA-DRB1* and *HLA-DQA1* genes, are associated with both MS risk and elevated anti-EBNA IgG levels.^4,5,6^ The possibility of confounding is suggested by studies showing that the increased MS risk associated with IM decreased in magnitude^7^ or became statistically nonsignificant^8^ after controlling for potential confounders, including genetic ancestry and HLA-DRB1*1501 carrier status. However, few studies have extensively addressed measured and unmeasured shared familial genetic and environmental factors as potential confounders that may be associated with both IM and MS. Therefore, methods such as within-sibling studies, which control for unmeasured shared familial factors (because members of the same family share important characteristics) by comparing siblings discordant for IM, are needed to better understand the relationship between IM and MS.

Adolescence may be an important period of susceptibility for the putative association of IM with MS pathogenesis compared with earlier childhood,^9,10^ consistent with associations for other risk factors for MS in adolescence: pneumonia and other infections,^11,12^ higher body mass index,^13^ concussion,^14^ and lower serum vitamin D levels.^15^ Infectious mononucleosis in early adulthood (20-24 years of age) has also been associated with increased MS risk, although the effect size is smaller than for IM in adolescence.^16^

This study used a large population–based birth cohort in Sweden to assess the association of hospital-diagnosed IM in childhood (from birth to 10 years of age) and adolescence (11-19 years of age) with risk of an MS diagnosis from 20 years of age. The association of IM in early adulthood (20-24 years of age) with a subsequent MS diagnosis was also assessed by treating IM as a time-dependent variable to ensure that IM predates the MS diagnosis. Cox proportional hazards regression stratified by family to compare siblings (within-sibling analysis) was used to account for measured and unmeasured shared familial factors. If the association between IM and MS is confounded by shared familial factors, the magnitude of estimates and statistical significance would be attenuated when results from within-sibling analyses are compared with conventional analyses.

## Methods

### Study Population

This population-based cohort, which was identified by the Total Population Register, comprised all individuals born in Sweden from January 1, 1958, to December 31, 1994, who reached 25 years of age from January 1, 1990, to December 31, 2019, with both parents also alive in 1990 to facilitate identification of all first-degree relatives and identify MS diagnoses in parents. Participants aged 20 years were followed up from January 1, 1978, to December 31, 2018, with a median follow-up of 15.38 (IQR, 8.68-23.55; range, 0.01-40.96) years. The unique individual Swedish personal identification number was used for data linkage across various Swedish registers to identify cohort members’ hospital-based diagnoses and their first-degree relatives (eMethods in the Supplement).^17,18^ The analysis was restricted to the youngest generation (including familial information on parents), because most parents, their siblings, and grandparents and their siblings were born at an earlier period when register coverage was too incomplete to adequately capture information on IM in childhood among them. A total of 2 492 980 individuals were included in this study (Figure). Ethical approval for this study was received from the Swedish Ethical Review Authority. Informed consent was not required by the authority because the study was based only on anonymized register data. This study followed the Strengthening the Reporting of Observational Studies in Epidemiology (STROBE) reporting guideline for cohort studies.

**Figure.**
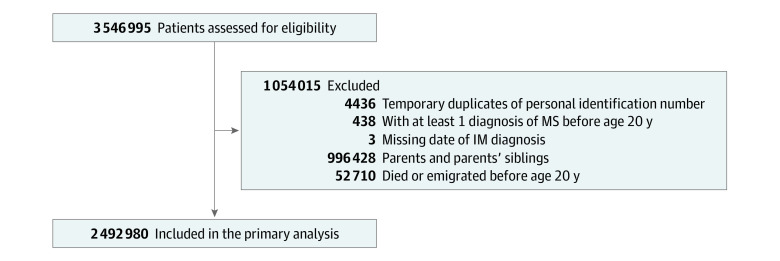
Flowchart of Participant Selection Temporary duplicates are due to the assignment of a previously deceased person's personal identification number to some immigrants to Sweden. Parents, their siblings, and grandparents and their siblings were not members of the analysis cohort because register coverage was more incomplete during their childhood. IM indicates infectious mononucleosis; MS, multiple sclerosis.

### Outcome

A diagnosis of MS from 20 years of age was identified for members of the index cohort or at any age for parents using *International Classification of Diseases, Eighth Revision* (*ICD-8*) or *International Classification of Diseases, Ninth Revision* (*ICD-9*) code 340 and *International Statistical Classification of Diseases and Related Health Problems, Tenth Revision* (*ICD-10*) code G35. To ensure diagnostic accuracy, 2 MS diagnoses recorded at a minimum of 6 months apart were required, which makes exclusion of misdiagnoses and relevant differential diagnoses more likely.^19^ Validation studies have shown a high level of accuracy both for MS diagnoses (positive predictive value of 92.50%)^20^ and when using at least 2 MS diagnoses to identify MS (positive predictive value and sensitivity >0.90%).^21^ The first diagnosis was used to define date of first MS diagnosis. Individuals with only 1 MS diagnosis or with separate MS diagnoses recorded within 6 months were not considered to have confirmed MS (n = 920).

### Exposure and Potential Confounding Factors

Primary or secondary diagnoses of IM before 25 years of age were identified using *ICD-8* or *ICD-9* code 075 and *ICD-10* code B27 (EBV titer data were not available). To examine the age-defined periods of potential susceptibility for the association of IM with risk of an MS diagnosis, IM diagnoses before 25 years of age were categorized as occurring in childhood (from birth to 10 years of age), in adolescence (11-19 years of age), or in early adulthood (20-24 years of age).^9,10,16^ Sex, an MS diagnosis in either parent, birth order (first, second, third, or fourth or subsequent), and maternal and paternal age at birth of the cohort member (the difference between cohort member’s date of birth and parents’ date of birth divided by 365.25) were considered potential confounding factors.

### Statistical Analysis

Data were analyzed from October 2020 to July 2021. There was no missing information for outcome and confounding factors. Three individuals who had missing information for date of IM diagnosis were excluded (Figure). STATA, version 16.0 (StataCorp LLC), was used for data analysis. Conventional Cox proportional hazards regression was used to test the association between IM in childhood or adolescence and the risk of an MS diagnosis from 20 years of age. The association of IM in early adulthood with a subsequent MS diagnosis was also examined. Follow-up was from 20 years of age until the first diagnosis of MS, death, first emigration, or study end (December 31, 2018), whichever occurred first. Cohort members who emigrated were censored on the date of first leaving Sweden and could not reenter the cohort to ensure no gaps in follow-up. We split follow-up time before and after the date of an IM diagnosis in early adulthood to model early adulthood IM diagnosis as a time-dependent covariate (ensuring we only considered associations with subsequent MS diagnoses). We adjusted for sex, parental MS diagnosis, birth order, and parental age at birth. Second-degree fractional polynomials suggested that parental age at birth was linearly associated with risk of an MS diagnosis. Violations of the proportional hazards assumption were detected for paternal age at birth, a parental MS diagnosis, and birth order using Schoenfeld residuals. However, the effect of violation was minimal based on scaled Schoenfeld residuals plotted against time (eFigure in the Supplement). Thus, results from analyses assuming proportional hazards are reported herein. Further analyses subdivided age categories for IM into 5-year periods and considered first MS diagnosis from 20 to 30 years of age or after 30 years of age (eTable in the Supplement).

To adjust for measured and unmeasured familial factors through estimating a cluster (sibling-set)–specific baseline hazard, stratified Cox proportional hazards regression was used for within-sibling analysis where the mother’s identification number was the stratification variable. The follow-up time and variables included in the analysis were the same as those described above. The magnitude of shared familial confounding was measured as the attenuation of estimates from Cox proportional hazards regression models stratified by family, compared with conventional Cox proportional hazards regression. For the stratified Cox proportional hazards regression, fractional polynomials suggested that parental age at birth was linearly associated with risk of an MS diagnosis. Violation of the proportional hazards assumption was detected for birth order using Schoenfeld residuals. Again, the effect of violation was minimal based on scaled Schoenfeld residuals plotted against time. Thus, results from analyses assuming proportional hazards were reported. Huber-White SEs were used to account for clustering within families.

A total of 5 sensitivity analyses using stratified Cox proportional hazards regression were performed. Sensitivity analysis 1 used a first MS diagnosis from 25 years of age to reduce possible bias because MS may have a relatively long prodromal period. Sensitivity analysis 2 excluded individuals who were born after 1987 to examine the potential influence of the incomplete national coverage of the Swedish Patient Register until 1987 on the association between IM and risk of an MS diagnosis. Sensitivity analysis 3 only included families with the same mother and father for all the children (within-full-sibling analysis). Sensitivity analysis 4 used the first diagnosis of any demyelinating disease of the central nervous system (CNS) to define the onset date of MS (individuals with a demyelinating disease–defined onset of MS before 20 years of age were excluded) as follows: MS, acute disseminated encephalomyelitis, optic neuritis, other acute disseminated demyelination, and other demyelinating disease of the CNS (*ICD-8* or *ICD-9* codes 323, 340, and 341 and *ICD-10* codes G04, H46, G35, G36, and G37).^22^ Acute disseminated encephalitis could not be identified in our data because *ICD* code precision of 4 digits is required, which was not available; therefore, encephalomyelitis diagnoses (*ICD-8* or *ICD-9* code 323 and *ICD-10* code G04) were exclusion criteria because they include acute disseminated encephalitis. Sensitivity analysis 5 included families with either parent alive in 1990 to address potential selection bias.

## Results

### Cohort Characteristics

A total of 2 492 980 individuals (1 312 119 men [52.63%] and 1 180 861 women [47.37%]; 1 290 007 first born [51.75%]; 26 954 [1.08%] with IM before 25 years of age; and 17 315 [0.69%] with either parent having an MS diagnosis) were included in this population–based cohort study (Table 1). Of these, 5867 individuals (0.24%) had an MS diagnosis from 20 years of age, at a median age of 31.50 (IQR, 26.78-37.54; range, 20.01-58.93) years. Results from stratified Cox proportional hazards regression indicated that being female (hazard ratio [HR], 2.62; 95% CI, 2.44-2.81) and older maternal age at birth (HR for each additional year, 1.07; 95% CI, 1.04-1.11) were associated with increased risk of an MS diagnosis; later-born children were at lower risk of being diagnosed with MS compared with first-born children (HR for second born, 0.91 [95% CI, 0.83-0.99]; HR for third born, 0.81 [95% CI, 0.68-0.97]; HR for fourth born or subsequent birth, 0.67 [95% CI, 0.49-0.91]) (Table 1); paternal age at birth was not associated with an increased risk of an MS diagnosis (HR, 0.98; 95% CI, 0.96-1.01) (Table 1). As expected, the increased risk associated with either parent having an MS diagnosis using an adjusted conventional Cox proportional hazards regression model (HR, 8.04; 95% CI, 7.16-9.04) became statistically nonsignificant after controlling for unmeasured shared familial factors using stratified Cox proportional hazards regression (HR, 1.95; 95% CI, 0.37-10.27).

**Table 1.** Characteristics of the Birth Cohort Stratified by MS With HRs for Risk of MS Diagnosis

Characteristic	MS diagnosis^a^		Cox proportional hazards regression, HR (95% CI)		
	Absent (n = 2 487 113)	Present (n = 5867)	Conventional		Stratified adjusted^d^
			Unadjusted^b^	Adjusted^c^	
Sex					
Male	1 310 269 (52.68)	1850 (31.53)	1 [Reference]	1 [Reference]	1 [Reference]
Female	1 176 844 (47.32)	4017 (68.47)	2.56 (2.42-2.70)	2.55 (2.41-2.69)	2.62 (2.44-2.81)
Age at birth, mean (SD), y^e^					
Maternal	27.62 (5.05)	27.36 (5.10)	1.01 (1.01-1.02)	1.00 (1.00-1.01)	1.07 (1.04-1.11)
Paternal	30.48 (5.88)	30.27 (5.84)	1.01 (1.01-1.01)	1.01 (1.00-1.01)	0.98 (0.96-1.01)
Parental MS diagnosis					
Neither parent	2 470 095 (99.32)	5570 (94.94)	1 [Reference]	1 [Reference]	1 [Reference]
Either parent	17 018 (0.68)	297 (5.06)	8.00 (7.12-8.99)	8.04 (7.16-9.04)	1.95 (0.37-10.27)
Birth order					
First	1 286 766 (51.74)	3241 (55.24)	1 [Reference]	1 [Reference]	1 [Reference]
Second	861 430 (34.64)	2014 (34.33)	1.10 (1.04-1.17)	1.06 (1.00-1.12)	0.91 (0.83-0.99)
Third	269 191 (10.82)	512 (8.73)	1.15 (1.04-1.26)	1.08 (0.98-1.19)	0.81 (0.68-0.97)
Fourth or subsequent	69 726 (2.80)	100 (1.70)	1.15 (0.94-1.41)	1.05 (0.86-1.29)	0.67 (0.49-0.91)
IM, birth to 10 y					
No	2 483 029 (99.84)	5851 (99.73)	1 [Reference]	1 [Reference]	1 [Reference]
Yes	4084 (0.16)	16 (0.27)	1.85 (1.13-3.02)	1.98 (1.21-3.23)	2.87 (1.44-5.74)
IM, 11-19 y					
No	2 469 259 (99.28)	5759 (98.16)	1 [Reference]	1 [Reference]	1 [Reference]
Yes	17 854 (0.72)	108 (1.84)	3.27 (2.70-3.95)	3.00 (2.48-3.63)	3.19 (2.29-4.46)
IM, 20-24 y					
No	2 482 238 (99.80)	5850 (99.71)	1 [Reference]	1 [Reference]	1 [Reference]
Yes	4875 (0.20)	17 (0.29)	1.69 (1.05-2.72)	1.89 (1.18-3.05)	1.51 (0.82-2.76)

Abbreviations: HR, hazard ratio; IM, infectious mononucleosis; MS, multiple sclerosis.

^a^ Unless otherwise indicated, data are expressed as number (%) of participants.

^b^ The association between each variable in the table and risk of an MS diagnosis was examined separately.

^c^ All variables in the table were included.

^d^ Indicates within-sibling analysis in which the mother’s identification number was the stratification variable; all variables in the table were included.

^e^ Maternal age at birth was highly correlated with paternal age at birth (*r* = 0.73). To account for potential collinearity, parental ages at birth were included in 2 separate stratified Cox proportional hazards regression models, where model 1 included maternal age at birth and the rest of the variables listed (excluding paternal age) and model 2 included paternal age at birth and the rest of the variables listed (excluding maternal age). The HRs for maternal age and paternal age at birth were 1.06 (95% CI, 1.04-1.08) and 1.03 (95% CI, 1.01-1.05), respectively.

### IM and Risk of an MS Diagnosis

Compared with those without IM, individuals with IM in childhood and adolescence were at a greater risk of being diagnosed with MS using an adjusted conventional Cox proportional hazards regression model (HRs, 1.98 [95% CI, 1.21-3.23] and 3.00 [95% CI, 2.48-3.63], respectively). This risk remained statistically significant and increased in magnitude after further controlling for unmeasured shared familial factors using stratified Cox proportional hazards regression (HRs, 2.87 [95% CI, 1.44-5.74] and 3.19 [95% CI, 2.29-4.46], respectively). Infectious mononucleosis in early adulthood was associated with an increased risk of an MS diagnosis using an adjusted conventional Cox proportional hazards regression model but at a lower magnitude than at younger ages (HR, 1.89; 95% CI, 1.18-3.05). This risk became statistically nonsignificant after controlling for unmeasured shared familial factors using stratified Cox regression (HR, 1.51; 95% CI, 0.82-2.76). The eTable in the Supplement shows a similar pattern of rising and then waning risk with age, with no attenuation of risk magnitude when follow-up begins after 30 years of age.

### Sensitivity Analyses

Results from sensitivity analyses are shown in Table 2. Restricting MS diagnoses to 25 years or older in sensitivity analysis 1 showed that the increased risk of being diagnosed with MS associated with IM in childhood and adolescence remained statistically significant and was not attenuated in magnitude (HRs, 3.72 [95% CI, 1.44-9.56] and 3.96 [95% CI, 2.65-5.90], respectively). Sensitivity analysis 2 found no substantial differences in HR, excluding or including individuals born after 1987. Sensitivity analysis 3 also found no substantial differences in HR between within-sibling analysis and within-full-sibling analysis. Sensitivity analysis 4 found that IM in childhood (HR, 3.24; 95% CI, 1.87-5.60) and adolescence (HR, 2.77; 95% CI, 2.08-3.68), but not in early adulthood, was associated with an increased risk of an MS diagnosis when the first demyelinating diseases of the CNS diagnosed from 20 years of age were defined as the possible onset of MS (and after exclusion of anyone with a demyelinating disease diagnosis before 20 years of age). Sensitivity analysis 5 found no substantial differences in HR when excluding or including families with only 1 parent alive in 1990.

**Table 2.** Adjusted HRs for Risk of MS Diagnosis From Sensitivity Analyses

Age at IM diagnosis	Stratified Cox proportional hazards regression, HR (95% CI)^a^				
	Sensitivity analysis 1^b^	Sensitivity analysis 2^c^	Sensitivity analysis 3^d^	Sensitivity analysis 4^e^	Sensitivity analysis 5^f^
Birth to 10 y					
No	1 [Reference]	1 [Reference]	1 [Reference]	1 [Reference]	1 [Reference]
Yes	3.72 (1.44-9.56)	2.60 (1.23-5.50)	2.68 (1.32-5.45)	3.24 (1.87-5.60)	2.90 (1.45-5.80)
11-19 y					
No	1 [Reference]	1 [Reference]	1 [Reference]	1 [Reference]	1 [Reference]
Yes	3.96 (2.65-5.90)	3.45 (2.34-5.08)	3.45 (2.43-4.89)	2.77 (2.08-3.68)	3.26 (2.32-4.57)
20-24 y					
No	1 [Reference]	1 [Reference]	1 [Reference]	1 [Reference]	1 [Reference]
Yes	1.89 (0.99-3.62)	1.51 (0.80-2.83)	1.76 (0.97-3.22)	1.48 (0.91-2.38)	1.53 (0.85-2.72)

Abbreviations: HR, hazard ratio; IM, infectious mononucleosis; MS, multiple sclerosis.

^a^ All variables listed in the table and parental ages at birth, a parental MS diagnosis, birth order, and sex were included in the sensitivity analyses.

^b^ Uses a first MS diagnosis from 25 years of age to further exclude the possibility of reverse causation.

^c^ Excludes individuals who were born after 1987 to examine the influence of the incomplete national coverage of the National Patient Register until 1987 on the association between IM and risk of an MS diagnosis.

^d^ Only includes families with the same mother and father for all of the children (within full sibling analysis).

^e^ Uses the first demyelinating diseases of the central nervous system diagnosed from 20 years of age as possible onset of MS.

^f^ Includes families with either parent still alive in 1990. Because maternal age at birth was highly correlated with paternal age at birth (*r* = 0.73), maternal age at birth data missing for families with only the father still alive in 1990 (1.46% of the population included) was replaced by the paternal age at birth. Similarly, paternal age at birth data missing for families with only the mother still alive in 1990 (5.14% of the population included) was replaced by the maternal age at birth. Parental MS diagnosis was only for either the mother’s or the father’s diagnosis for families with only the mother or the father still alive in 1990 (6.60% of population included).

## Discussion

Hospital-diagnosed IM in childhood and most notably in adolescence was associated with increased risk of a subsequent MS diagnosis, independent of measured and unmeasured shared familial factors addressed by stratified Cox proportional hazards regression. There was less evidence of an independent association of MS with IM in early adulthood. This age-defined pattern of risk may reflect variation in susceptibility to environmental exposures due to developmental changes of the immune system and CNS. Because MS may have an asymptomatic or prodromal period of approximately 5 to 10 years before clinical onset, a diagnosis of IM in adolescence could potentially signal that IM is a consequence of prodromal MS.^23^ Allowing a delay of at least 5 years between IM and MS (with evidence of even longer intervals between IM and MS in further sensitivity analyses) helps to alleviate such concerns, because the magnitude of the estimates for the association of IM in adolescence with subsequent MS were not attenuated. Also, IM in early adulthood was not so notably associated with MS.

Infection with EBV in early childhood is common and typically asymptomatic, which reduces the possibility of experiencing subsequent IM owing to EBV, just as the risk of more severe manifestations of other infections increases with age (eg, for poliomyelitis).^24^ This is consistent with the association of having older siblings and reduced MS risk, because having older siblings may increase infection risk at an earlier age, thereby reducing risk of other immune-mediated diseases.^25,26^ We speculate that the intense immune activation associated with IM (rather than EBV infection per se) results in priming with possible recruitment of mimicry autoimmunity, thus increasing MS risk. We used hospital-diagnosed IM in this study, which is likely to identify more severe manifestations.

The waning of IM-associated risk for MS after adolescence is consistent with other research.^16^ Our results suggest that childhood and in particular adolescence seem to be periods when acute IM is relevant to MS risk. Such an age-specific pattern is consistent with prior research on other environmental risk factors, in which some exposures seem to have greater consequences in adolescence than in earlier childhood,^27^ including hospital-diagnosed infections other than EBV or IM.^12^

Mechanisms that potentially explain why acute IM (during age-defined periods of heightened susceptibility) is associated with increased risk of an MS diagnosis include the cross-activation of autoreactive T cells to the CNS due to the homologous sequences or structures shared between the EBV pathogens and myelin antigens. Three EBV proteins—the tegument protein BRRF2, EBNA-1, and the small capsid protein (BFRF3)—share epitopes with myelin basic proteins (eg, DLST and septin-9).^28,29^ Antibodies to BRRF2, EBNA-1, and BFRF3 cross-react with myelin antigens, which can lead to recurrent autoimmune CNS damage.^28,29^ Antibodies to EBNA-1 can also cross-react with the chloride-channel protein anoctamin 2 and are associated with increased MS risk.^30^ Indeed, a significantly increased presence of anti–viral capsid antigen IgG and anti-EBNA-1 IgG antibodies^31^ and significantly higher CD4^+^ and CD8^+^ T-cell responses to EBNA-1 have been reported in patients with MS compared with control individuals.^28^ The EBV-infected autoreactive B cells hypothesis may be another possible mechanism: EBV infects resting B cells and converts them into immortalized lymphoblastoid cell lines, resulting in the production of ENBA-1 through ENBA-6 and latent membrane proteins (LMP-1, LMP-2A, and LMP-2B).^32^ B cells can further transform into memory B cells because LMP-1 mimics CD40 signaling.^32^ The memory B cells can infiltrate the blood-brain barrier, then become activated and develop into antibody-secreting plasma cells triggered by interleukin 21,^33^ causing inflammation in the CNS and in turn increasing MS risk.^34^ B cells and plasmablasts/plasma cells are present in various parts of the CNS in patients with MS.^34,35^ Our findings lend further weight to the notion that EBV plays a role in pathogenesis, where the pattern of exposure and acute manifestation of the infection are relevant, rather than being a bystander phenomenon due to MS disease activity or susceptibility to MS resulting in a greater likelihood of IM.

Older maternal, rather than paternal, age at birth was another risk factor for MS diagnosis, which is inconsistent with prior research,^36^ possibly because maternal and paternal age are highly correlated (*r* = 0.73). When only paternal age or maternal age at birth were included in the adjusted stratified Cox proportional hazards regression, both were associated with an increased risk of an MS diagnosis. A diagnosis of MS in a parent conveyed a high magnitude of increased risk for MS, indicating the importance of genetic predisposition. As expected, this association was attenuated by stratified Cox proportional hazards regression that adjusted for shared familial factors.

### Strengths and Limitations

The strengths of this study include the use of a large population-based birth cohort with sufficient statistical power and prospective recording of IM before MS diagnosis. We also examined IM at 3 theoretically relevant developmental stages (childhood, adolescence, and early adulthood) to assess potential periods of susceptibility to EBV exposure. Importantly, stratified Cox proportional hazards regression was used to reduce confounding by shared familial factors invariant among each sibling cluster.

This study also has some limitations. Infectious mononucleosis diagnosed and treated in primary care could not be included, and hospital-based outpatient diagnoses were not available until 2001, likely resulting in selection of more severe acute manifestations of the infection. Some IM and MS diagnoses may be missing owing to the incomplete national coverage for inpatient registrations before 1987 and the unavailability of outpatient information before 2001, though our sensitivity analysis found no substantial differences in HRs between analyses including and excluding individuals who were born after 1987.

We could not assess MS onset earlier than the first MS diagnosis because the first symptomatic onset of MS could not be identified in the current study. However, excluding those with any demyelinating diseases before 20 years of age and extending the minimal interval between putative exposure and diagnosis did not suggest reverse causation. We could not adjust for vitamin D level, smoking, and body mass index. However, there are strong shared familial influences for body mass index, smoking, and vitamin D levels in childhood and adolescence,^37,38,39,40^ so the stratified Cox proportional hazards regression should have addressed this issue, at least in part. For complete family linkage and information from both parents, parents had to survive to at least 1990, thus possibly introducing selection bias. A sensitivity analysis based on only 1 surviving parent did not produce notably different results.

## Conclusions

The findings of this population–based cohort study suggest that IM in childhood and particularly adolescence is associated with a subsequent MS diagnosis, independent of shared familial factors. Therefore, greater susceptibility to infection is less likely to be the explanation.

## Notes

Supplement. **eMethods.** Study Population **eTable.** Adjusted Hazard Ratios for Risk of a Multiple Sclerosis Diagnosis With Infectious Mononucleosis in Smaller Age Categories and Follow-up for First Multiple Sclerosis Diagnosis at Different Ages **eFigure.** Scaled Schoenfeld Residuals Plotted Against Time for Paternal Age at Birth, a Parental MS Diagnosis, and Birth Order
eReferences
