# Supplementary material for: Association of Infectious Mononucleosis in Childhood and Adolescence With Risk for a Subsequent Multiple Sclerosis Diagnosis Among Siblings
Source: JAMA Netw Open. 2021 Oct 11;4(10):e2124932. doi: 10.1001/jamanetworkopen.2021.24932 (PMC8506233; doi:10.1001/jamanetworkopen.2021.24932)
Supplement: Supplement. — eMethods. Study Population eTable. Adjusted Hazard Ratios for Risk of a Multiple Sclerosis Diagnosis With Infectious Mononucleosis in Smaller Age Categories and Follow-up for First Multiple Sclerosis Diagnosis at Different Ages eFigure. Scaled Schoenfeld Residuals Plotted Against Time for Paternal Age at Birth, a Parental MS Diagnosis, and Birth Order eReferences [file jamanetwopen-e2124932-s001.pdf]

Supplementary Online Content

Xu Y, Hiyoshi A, Smith KA, et al. Association of infectious mononucleosis in childhood and adolescence with risk for a subsequent multiple sclerosis diagnosis among siblings. *JAMA Netw Open*. 2021;4(10):e2124932. doi:10.1001/jamanetworkopen.2021.24932

eMethods. Study Population

eTable. Adjusted Hazard Ratios for Risk of a Multiple Sclerosis Diagnosis With Infectious Mononucleosis in Smaller Age Categories and Follow-up for First Multiple Sclerosis Diagnosis at Different Ages

eFigure. Scaled Schoenfeld Residuals Plotted Against Time for Paternal Age at Birth, a Parental MS Diagnosis, and Birth Order

eReferences

This supplementary material has been provided by the authors to give readers additional information about their work.

© 2021 Xu Y et al. *JAMA Network Open*.

eMethods. Study Population

Two Swedish general population-based registers with high validity were used in the current study: the Total Population Register and the National Patient Register. The unique individual Swedish personal identification number was used for data linkage. The Total Population Register has been updated annually since 1968 and collects life events for the entire population of Sweden, including dates of birth, death, and migration.[1] The National Patient Register for inpatient care was established in 1964 and attained complete national coverage in 1987, with hospital-based outpatient diagnoses collected since 2001.[2] The National Patient Register contains data on all inpatient and outpatient diagnoses, admission, discharge, and procedures in hospital.[2]

© 2021 Xu Y et al. *JAMA Network Open*.

eTable. Adjusted Hazard Ratios for Risk of a Multiple Sclerosis Diagnosis With Infectious Mononucleosis in Smaller Age Categories and Follow-up for First Multiple Sclerosis Diagnosis at Different Ages

	Stratified Cox regression		
	Analysis-1<br>MS from age 20 years	Analysis-2<br>MS ages 20 to 30 years	Analysis-3<br>MS after age 30 years
IM, birth to age 5 years			
No	1 (reference)	1 (reference)	1 (reference)
Yes	1.39 (0.49-3.99)	1.24 (0.36-4.29)	2.13 (0.26-17.37)
IM, age 6-10 years			
No	1 (reference)	1 (reference)	1 (reference)
Yes	5.72 (1.93-16.96)	5.51 (1.16-26.17)	6.10 (1.38-26.93)
IM, age 11-15 years			
No	1 (reference)	1 (reference)	1 (reference)
Yes	3.45 (1.93-6.17)	2.17 (1.04-4.52)	7.50 (3.02-18.63)
IM, age 16-19 years			
No	1 (reference)	1 (reference)	1 (reference)
Yes	3.10 (2.09-4.60)	2.98 (1.77-5.00)	3.64 (1.95-6.79)
IM, age 20-24 years			
No	1 (reference)	1 (reference)	1 (reference)
Yes	1.50 (0.82-2.76)	0.97 (0.35-2.69)	2.60 (1.07-6.36)

© 2021 Xu Y et al. *JAMA Network Open*.

Abbreviations: IM = infectious mononucleosis. MS = multiple sclerosis

Hazard ratios and 95% confidence interval from stratified Cox regression (within-sibling analysis with the mother's identification number as the stratification variable), adjusted for sex, parental age at birth, parental multiple sclerosis diagnosis, and birth order, were reported here.

For analysis-1, follow-up was from age 20 years until the first diagnosis of MS, death, first emigration, or study end (31st December 2018), whichever occurred first. For analysis-2, follow-up was from age 20 until the first diagnosis of MS, death, first emigration, attaining the age of 30 years, or study end (31st December 2018), whichever occurred first. For analysis-3, follow-up was from after age 30 years until the first diagnosis of MS, death, first emigration, or study end (31st December 2018), whichever occurred first.

© 2021 Xu Y et al. *JAMA Network Open*.

eFigure. Scaled Schoenfeld Residuals Plotted Against Time for Paternal Age at Birth, a Parental MS Diagnosis, and Birth Order

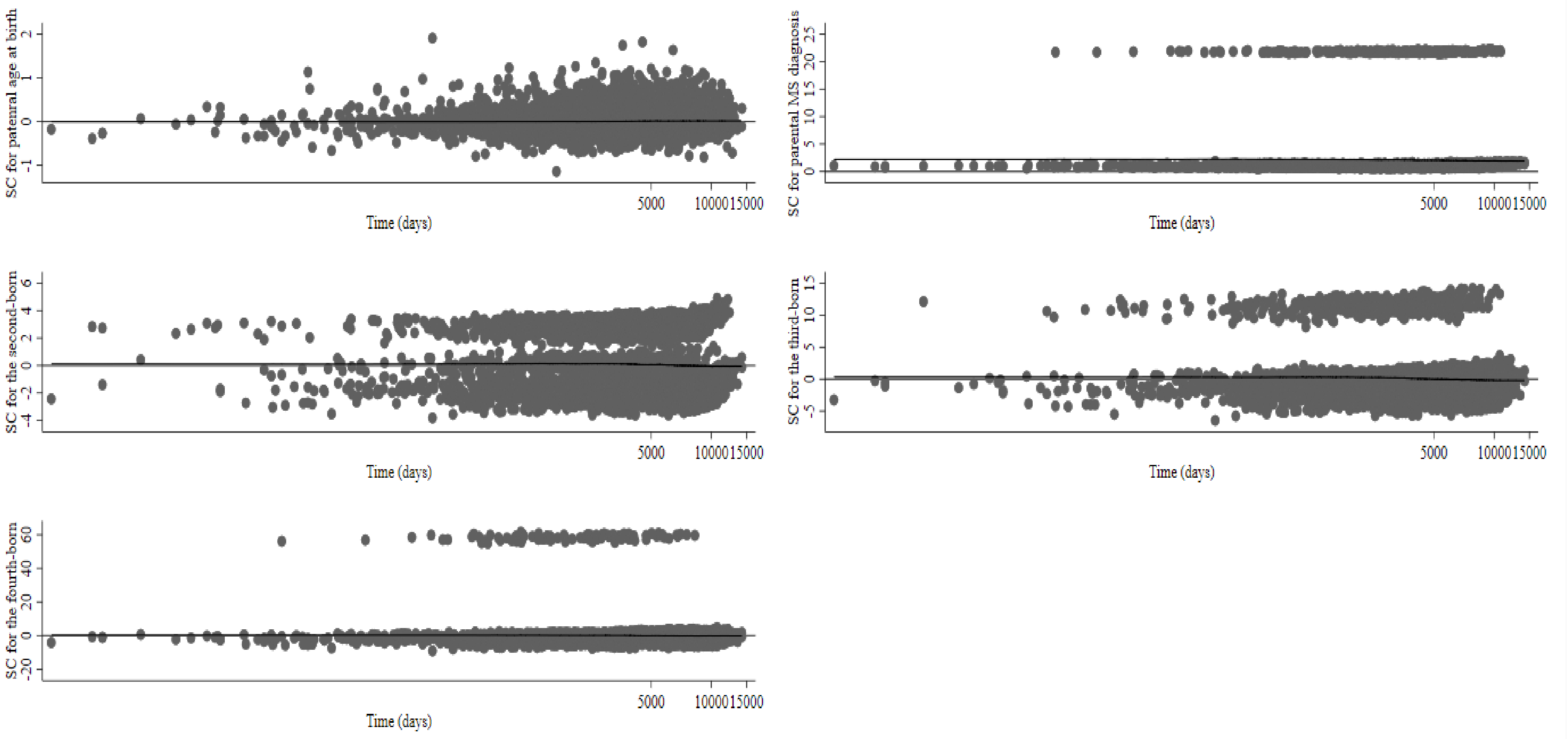

For birth order, the first-born was the reference group.
MS = multiple sclerosis; SC = Schoenfeld residuals.

© 2021 Xu Y et al. *JAMA Network Open*.

eReferences

1. Ludvigsson JF, Almqvist C, Bonamy AK, et al. Registers of the Swedish total population and their use in medical research. *Eur J Epidemiol*. 2016;31(2):125-136. doi:10.1007/s10654-016-0117-y
2. Ludvigsson JF, Andersson E, Ekbom A, et al. External review and validation of the Swedish national inpatient register. *BMC Public Health*. 2011;11(1):450. doi:10.1186/1471-2458-11-450

© 2021 Xu Y et al. *JAMA Network Open*.
